# Letter to the Editor Concerning the Publication: “Efficacy of First-Time Intragastric Balloon in Weight Loss: a Systematic Review and Meta-Analysis of Randomized Controlled Trials”

**DOI:** 10.1007/s11695-016-2485-5

**Published:** 2016-12-12

**Authors:** Mariusz Wylezol, Aleksandra Mojkowska

**Affiliations:** Department of Surgery, Military Institute of Aviation Medicine, Warsaw, Poland

Dear Sir

We have read with interest the study entitled “Efficacy of First-Time Intragastric Balloon in Weight Loss: a Systematic Review and Meta-analysis of Randomized Controlled Trials” [1].

Meta-analyses of randomized controlled trials are the most valuable source of our knowledge, because results can be generalized to a larger population. Usually, they are the background for practicing surgeons when deciding which type of treatment could be the best choice offered to the patient. For this reason, we are convinced that such studies should be performed on the basis on currently available methods of treatment and medical devices used today.

Therefore, we are surprised that the abovementioned study involves historical or experimental types of intragastric balloon which have not been applied for more than 20 years. We found that this concerns 8 studies from the total number of 20 studies included by the authors in their analysis (Table 1). What is also worth mentioning is that the population of patients included in the studies with historical devices accounts for 24% (*n* = 289) of the whole population of patients involved in the meta-analysis.

**Table 1:** Studies included in the meta-analysis with historical or experimental types of intragastric balloon

	Author	Year of publication	Population *n* = 289	Fulfillment	Balloon type
1.	Lindor [2]	1987	21	Air-filled	Garren-Edward gastric bubble
2.	Meshkinpour [3]	1988	23	Air-filled	Garren-Edward gastric bubble
3.	Benjamin [4]	1988	46	Air-filled	Garren-Edward gastric bubble
4.	Ramhamadany [5]	1989	24	Air-filled	Ballobes bubble
5.	Hogan [6]	1989	59	Air-filled	Garren-Edward gastric bubble
6.	Mathus-Vligen [7]	1990	56	Air-filled	Ballobes bubble
7.	Geliebter [8]	1991	40	Fluid-filled	Breast implant
8.	Rigaud [9]	1995	20	Air-filled	Ballobes bubble

Even a brief comparison of the characteristic of historical or experimental balloons and currently used balloons demonstrates the significant differences in their capacity, shape, and time of treatment (Table 2). All of these differences could influence the final weight loss, tolerance, and complications. For example, we would like to highlight that, in 1992, the Food and Drug Administration (FDA) withdrew approval for the gastric bubble because of significant complications and weight loss recidivism [10]. It is also interesting that in one of the included studies (Geliebter), the fluid-filled balloon was made from a breast implant!

**Table 2:** Characteristics of historical or experimental balloons and currently used balloons

Balloon type	Garren-Edward gastric bubble	Ballobes bubble	Breast implant	Orbera intragastric balloon	ReShape dual intragastric balloon	Heliosphere BAG
Years of use	1984–1992	1988–???	???	1991–now	2007–now	2004–now
Fulfillment	Air	Air	Fluid	Fluid	Fluid	Air
Capacity	200–220 mL	500 mL	300 mL	400–700 mL	900 mL	600–960 mL
Shape	Cylindrical	Oval	???	Spherical	Bi-lobal	Spherical
Treatment period	4 months	3–4 months	3 months	6 months	6 months	6 months

The authors also performed a comparison between fluid-filled and air-filled balloons. However, we should be aware that all types of air-filled balloons involved in the analysis were historical, among them the Garren-Edward gastric bubble and the Ballobes bubble. On the opposite side of the analysis were fluid-filled balloons which are used today (Orbera intragastric balloon, ReShape dual intragastric balloon). We would also mention that air-filled balloons are also in use (Heliosphere BAG [11]) nowadays; however, they also differ in shape, capacity, and treatment period from historical devices. In fact, the authors compared historical types of balloons with modern ones.

To conclude, we ask ourselves whether, on the basis of such an analysis, it is possible to decide which type of treatment could and should be offered to patients.
